# Exploration of Novel Immunological Terms in Lung Cancer With Large Populations: Implications for Immunotherapy

**DOI:** 10.3389/fimmu.2022.924498

**Published:** 2022-06-30

**Authors:** Yuanshan Yao, Jing Wang, Fuzhi Yang, Wen Gao

**Affiliations:** Department of Thoracic Surgery, Shanghai Key Laboratory of Clinical Geriatric Medicine, HuaDong Hospital Affiliated to Fudan University, Shanghai, China

**Keywords:** NSCLC, immune terms, IRS signature, prognosis, immunotherapy

## Abstract

**Background:**

Ideal biomarkers to predict the response to immunotherapy in lung cancer are still lacking. Therefore, there is a need to explore effective biomarkers in large populations.

**Objective:**

The objective of this study is to explore novel immunological classifications that are associated with immunotherapy response through the ssGSEA algorithm.

**Methods:**

Six independent lung cancer cohorts were collected for analysis including The Cancer Genome Atlas (TCGA), Gene Expression Omnibus (GEO), and the EMBL-EBI database. The ssGSEA algorithm was performed to extract immune terms. Then, TCGA samples were involved as a training group and other cohorts were used as a validation group. After LASSO and Cox regression, prognostic associated immune terms were extracted and an immune-related risk score (IRS) signature was constructed. Furthermore, the association between IRS signature and clinical data, genome features, stemness indices analysis, tumor immune microenvironment, immunotherapy efficiency, and targeted therapy response was also investigated.

**Results:**

A total of 1,997 samples were enrolled in this study including six large lung cancer cohorts. Fifty-four immune terms were calculated through the ssGSEA algorithm in combined cohorts. Then, a nine-immune-term risk score model named IRS signature was established to predict the prognosis in combined cohorts. We classified patients into high-risk and low-risk subgroups according to the cutoff point. Subsequently, analysis of clinical data and genome features indicated that the patients in the high-IRS group tend to have advanced clinical features (clinical stage and T classification), as well as a higher level of copy number variation burden, higher tumor burden mutation, and higher tumor stemness indices. Immune landscape analysis demonstrated that high-IRS groups exhibited lower immune cell infiltration and immune-suppressive state. More importantly, the predicted result of the Tumor Immune Dysfunction and Exclusion analysis showed that high-IRS groups might be more insensitive to immunotherapy. Meanwhile, we have also identified that high-IRS groups were associated with better efficiency of several targeted drugs.

**Conclusion:**

To summarize, we identified a novel IRS model based on nine immune terms, which was quantified by the ssGSEA algorithm. This model had good efficacy in predicting overall survival and immunotherapy response in non-small cell lung cancer patients, which might be an underlying biomarker.

## Introduction

Nowadays, immunotherapy has significantly improved the prognosis of advanced non-small cell lung cancer (NSCLC) (1). Although PD1/L1 inhibitors have changed the treatment landscape of NSCLC, the optimal biomarker to predict the clinical response of immunotherapy is still lacking (2). PD-L1 expression level and tumor mutational burden (TMB) in tumor specimens are two reliable biomarkers in clinical practice so far (3). However, a proportion of PD-L1-negative patients can also benefit from PD1/L1 inhibitors. Thus, it is urgent to explore other effective biomarkers with greater accuracy.

Recently, there are various biomarkers to predict immunotherapy response to lung cancer patients derived from the tumor immune microenvironment, molecular alterations, and serum indexes (4). For example, tumor-infiltrating lymphocytes (T cells and B cells) and the spatial location of these immune cells were already validated to be associated with the benefit of immunotherapy (5, 6). However, controversy still exists. Colt et al. demonstrated that penetration of immune cells into the cancer cells represented a better prognosis compared to those in tumor stroma (7). Nevertheless, Paul et al. revealed that immune cells that were located at the tumor margin exhibited a better response to immunotherapy than those in the stroma or intra-tumor (8). In addition, the prognosis of patients who received immune checkpoint inhibitors was associated with a non-invasive source of pre-treatment serum NLR (neutrophil-to-lymphocyte ratio) (9, 10). Currently, there is still no single satisfactory biomarker to evaluate the efficacy of immunotherapy. Incorporating multiple methods may be the best way (11).

In recent years, immune-related genes have also attracted attention. T cell-inflamed gene expression profile, which consisted of 18 IFN-r associated genes, was also explored and confirmed to exert its clinical prediction value in various types of malignancies (12). Johnson et al. demonstrated that patients with higher HLA-DR obtained better clinical response, prognosis, and progression-free survival compared with lower HLA-DR patients (13). Nevertheless, little is known about the correlation between immune terms and immunotherapy response in NSCLC patients.

Meanwhile, two atypical patterns of treatment responses are particularly correlated with immunotherapy including pseudoprogression and hyperprogression, which occupy a certain amount of patients accepting immunotherapy (14). The underlying molecular mechanism remains unknown so far. In clinical applications even using the somewhat improved immune RECIST (iRECIST) criteria, it is probable that the treatment effect of several patients is misjudged, which could lead to loss of optimal treatment opportunities (15). Patients with improved or stable clinical symptoms may predict effective immunotherapy. Future work is warranted to refine imaging response criteria and explore potential biomarkers that can make treatment recommendations more clear and standardized.

In this study, we collected the expression data from the publicly accessible dataset with 1,997 NSCLC patients. Then, 54 immune terms were extracted through single-sample gene set enrichment analysis (ssGSEA). We identified nine immune terms with prognostic values and constructed an immune-related risk score (IRS) signature. Moreover, the IRS signature can be used as a biomarker to predict the efficacy of immunotherapy. Meanwhile, several targeted drugs were potential candidates for targeting this IRS signature. We hope that our study can provide a reference for the treatment of NSCLC.

## Materials and Methods

### Data Collection and Preprocessing

Six cohorts with complete clinical information and available expression matrix data were collected (TCGA, GSE37745, GSE50081, GSE68465, GSE73403, and E-GEOD-30219) after a comprehensive search in this study. TCGA-LUAD data and TCGA-LUSC data were retrieved from The Cancer Genome Atlas (TCGA) database (https://portal.gdc.cancer.gov/). In detail, the expression profile data from TCGA were originally in “FPKM” form and then converted to “TPM” form for higher comparability with microarray data. E-GEOD-30219 data were retrieved from the ArrayExpress database (https://www.ebi.ac.uk/arrayexpress/). In addition, four individual Gene Expression Omnibus (GEO) datasets were identified from the GEO dataset (https://www.ncbi.nlm.nih.gov/geo/), including GSE37745 (GPL570), GSE50081 (GPL570), GSE68465 (GPL96), and GSE73403 (GPL6480). Three packages in the R environment, namely, sva, cluster, and oompaBase, were used to combine data and reduce the likelihood of batch effects and magnitude harmonization. The codes used were uploaded in the figshare website (https://figshare.com/articles/software/Code/19995041).

### Single-Sample Gene Set Enrichment Analysis and Pathway Enrichment Analysis

The R package “clusterProfiler” was used to perform gene set enrichment analysis (GSEA), Gene Oncology (GO), and Kyoto Encyclopedia of Genes and Genomes (KEGG) analysis. The R package GSVA was used to conduct ssGSEA to evaluate the enrichment scores of 54 immune terms. The Hallmark gene set (MSigDB) was selected as a reference set to explore the difference in the oncogenetic pathways. Immune-related features used for ssGSEA quantification were collected from Genomic Data Commons (https://gdc.cancer.gov/about-data/publications/panimmune) and previous studies, which was available at figshare (https://figshare.com/articles/dataset/Untitled_Item/19368023).

### Calculation of Immune-Related Risk Score Signature for the Combined Patient Cohort

After removing batch effects, TCGA-LUAD and TCGA-LUSC cohorts were selected as the training cohort and other datasets were used as the validation cohort (GSE37745, GSE50081, GSE68465, GSE73403, and E-GEOD-30219). For the training cohort, univariate Cox analysis was performed to identify prognosis-related immune terms with the criteria *p*-value <0.05. Then, L1-penalized (LASSO) estimation across 1,000 iterations was used for dimension reduction. Immune terms with a frequency higher than 50 times were then selected for multivariate Cox regression analysis and IRS calculation using the following formula: Immune risk score = term1*coef1 + term2*coef2 + term3*coef3 + … + termN*coefN. R packages “SimDesign” and “tdROC” were used to get the best cutoff value of risk score in training and validation cohorts. The R packages “survival” and “survivalROC” were used to assess the prognostic value of the signature through Kaplan–Meier survival curve and the ROC curve.

### Features of Tumor Genomics Between Two IRS Groups

TMB means the number of mutations per megabase (mt/Mb). Somatic mutation data retrieved from the cBioPortal website (http://www.cbioportal.org/datasets, Lung Adenocarcinoma/Lung Squamous Cell Carcinoma, TCGA, PanCancer Atlas) was used to calculate TMB. The R package “maftools” was used to analyze significantly mutated genes with *p*-value < 0.05 between the two IRS groups and the interaction effect of gene mutations. GISTIC_2.0 (https://cloud.genepattern.org) was applied for copy number variation (CNV) analysis. Based on the output files from GISTIC_2.0, copy number gain burden and loss burden at the focal and arm levels were calculated. One-class logistic regression machine learning (OCLR) algorithm was used to quantify the tumor stemness index, including mRNAsi, mDNAsi, EREG-mRNAsi, and EREG-mDNAsi, whose process is available on https://bioinformaticsfmrp.github.io/PanCanStem_Web/. ESTIMATEScore, StromalScore, and ImmuneScore of each sample were calculated using the R package “ESTIMATE”. A higher score represents a more considerable amount of the corresponding lower tumor purity and higher stromal and immune cells in TMB.

### Prediction of Immunotherapeutic and Chemotherapeutic Response

The Tumor Immune Dysfunction and Exclusion (TIDE, http://tide.dfci.harvard.edu) algorithm was used to predict patient immunotherapy response. Each patient with a TIDE score <−0.2 was defined as a Responder, and a patient with a TIDE score >0.2 was defined as a No-responder. Subclass mapping algorithm (https://cloud.genepattern.org/) was used to assess similarities in response to immunotherapies between the 47 patients who responded to immunotherapies and two lung cancer patients from the IRS group. The response to chemotherapy drugs of patients was predicted based on the public pharmacogenomics database Genomics of Drug Sensitivity in Cancer (GDSC, https://www.cancerrxgene.org/), and the R package “pRRophetic” was used to estimate the half-maximal inhibitory concentration (IC_50_) of twelve commonly used chemo drugs for predicting the sensitivity of chemotherapy drugs.

### Statistical Analysis

All statistical analyses were performed with R version 4.0.2 and its appropriate packages. All statistics were two-sided and statistical significance was defined as *p*-value < 0.05. Data were analyzed with standard statistical tests as appropriate. Spearman correlation was used to estimate the correlations between continuous variables. Independent sample *t*-test was used to compare continuous variables with normal distribution. Wilcoxon rank-sum test was used to compare continuous variables with skewed distribution.

## Results

### Immune Terms’ Quantification

**Figure 1:**
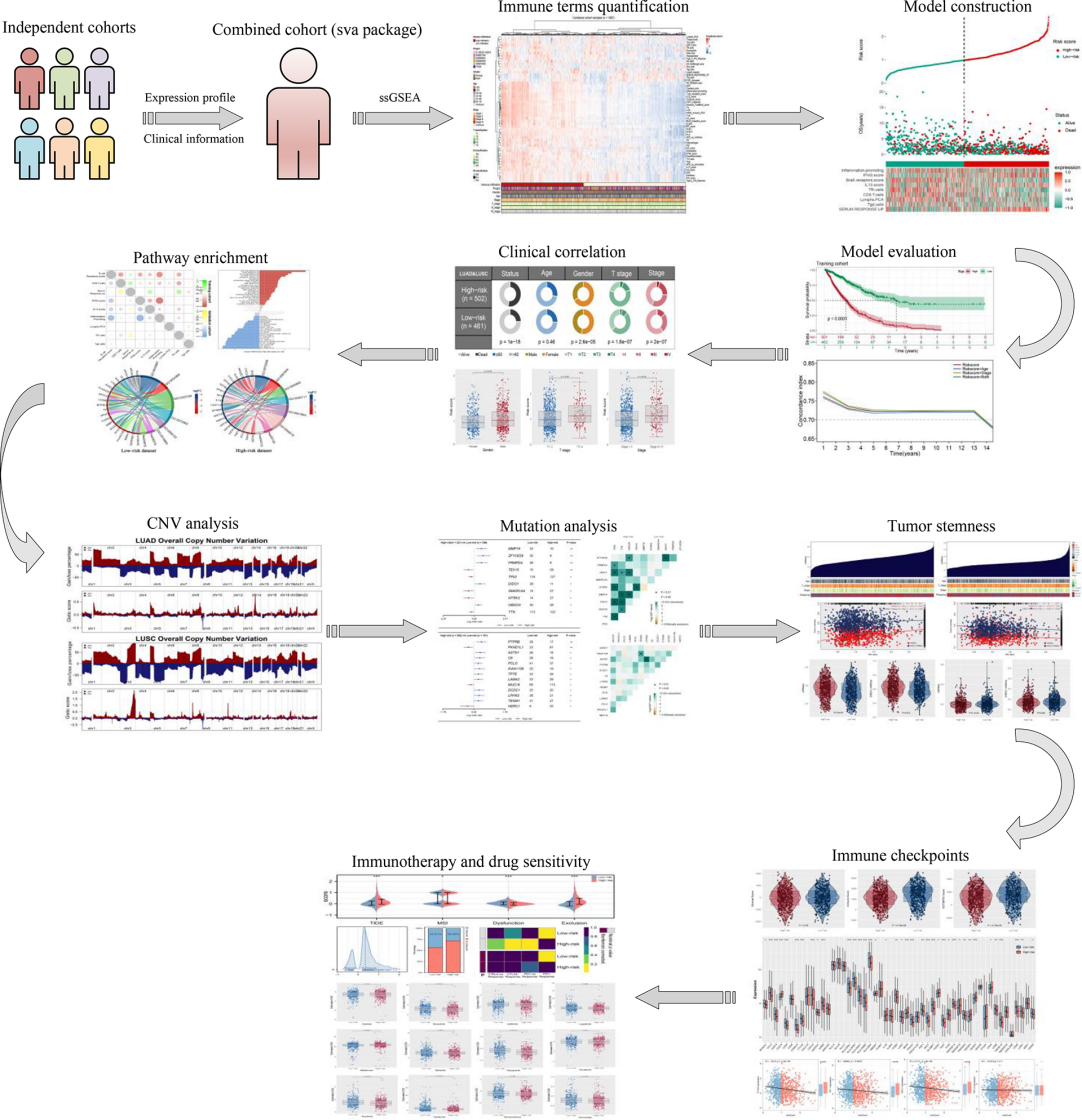
The flowchart of the whole study was shown in **Figure 1**.

**Figure 2:**
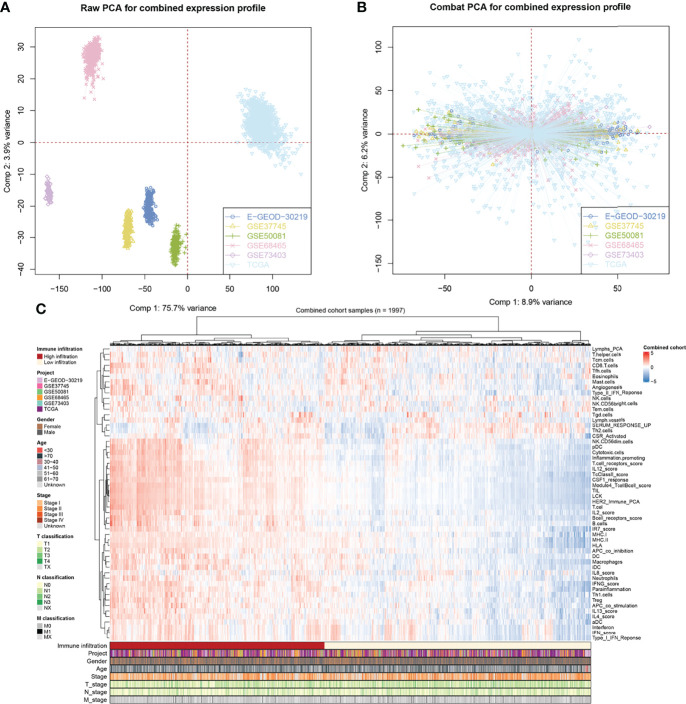
Combination of NSCLC cohort and quantification of immune terms. **(A, B)** The sva package was used for reduce the batch difference of individual cohort, including TCGA, GSE73403, GSE68465, GSE50081, GSE37745 and E-GEOD-30219; **(C)** ssGSEA algorithm was used to quantify the 53 immune terms based on the transcript data.

The flowchart of the whole study is shown in **Figure 1**. After a comprehensive search of the public database, six individual NSCLC cohorts met our criteria and were finally included in our analysis (TCGA, GSE73403, GSE68465, GSE50081, GSE37745, and E-GEOD-30219). A significant batch difference was observed between these cohorts (**Figure 2A**, comp1: 75.7% variance, comp2: 3.9% variance). Then, the sva package was used to remove the batch effect of these six NSCLC cohorts (**Figure 2B**, comp1: 8.9% variance, comp2: 6.2% variance). The combined expression profile was then used for the 54 immune terms’ quantification based on the ssGSEA algorithm, which is shown in **Figure 2C**.

### Identification of the Prognosis-Related Immune Terms

**Figure 3:**
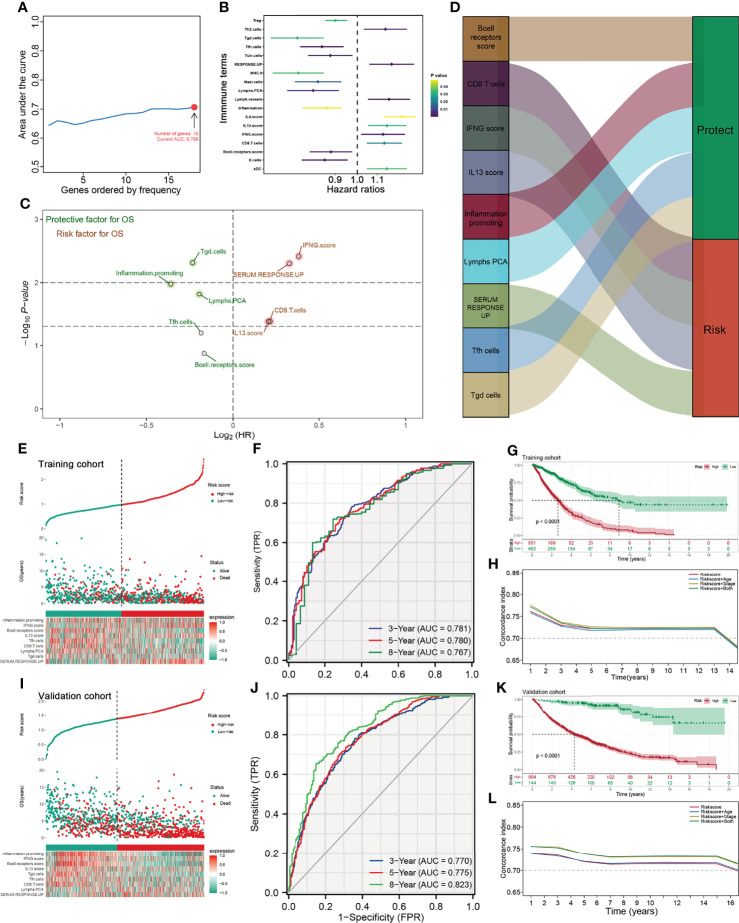
Identification the immune terms significantly associated with patients prognosis. **(A)** Iterative LASSO regression was used for high-frequency features selection and 18 prognosis-related terms were retained; **(B)** Univariate Cox analysis was performed to identified prognosis-related immune terms with the threshold of P < 0.05; **(C, D)** Multivariate Cox analysis was performed to identify immune terms for prognosis-model construction; **(E)** Risk plot showed a higher percentage of dead cases in the high IRS group (training cohort); **(F)** ROC curve showed a satisfactory prediction efficiency of prognosis model; **(G)** Kaplan-Meier survival curve showed that high IRS patients might have a worse OS; **(H)** Time-dependent ROC curve showed that IRS signature has stable predictive ability of OS in a different time (training cohort); **(I–L)** The risk plot, ROC curve, Kaplan-Meier survival curve and Time-dependent ROC curve in validation cohort.

To further identify the immune terms associated with patient prognosis, we first performed a univariate Cox analysis with the threshold of *p* < 0.05. Then, iterative LASSO regression was used for high-frequency features selection and 18 prognosis-related terms were retained (**Figures 3A, B**). Nine immune terms were selected for prognosis model construction based on the multivariate Cox analysis, including B-cell receptor score, CD8 T cells, IFNG score, IL13 score, Inflammation promoting, Lymphs PCA, SERUM RESPONSE UP, Tfh cells, and Tgd cells (**Figure 3C**). Among these, IFNG score, SERUM RESPONSE UP, CD8 T cells, and IL13 score were risk factors, yet Tgd cells, Inflammation promoting, Lymphs PCA, Tfh cell, and B-cell receptor score were protective factors (**Figure 3D** and **Figure S1A**). The model IRS was calculated with the following formula: “IRS = B cell receptor score * −0.115 + CD8 T cell * 0.148 + SERUM RESPONSE UP * 0.225 + IFNG score * 0.265 + IL13 score * 0.142 + Inflammation promoting * −0.249 + Lymphs PCA * −0.135 + Tfh cells * −0.128 + Tgd cells * −0.161”. SimDesign and the tdROC package were used to calculate the optimum cutoff values, and the result showed that the 0.98 and 1.38 were the best cutoff of training cohort and validation cohort, respectively (**Figures S1B,C**). In the training cohort, more death cases could be observed in the high-risk group (**Figure 3E**). The ROC curve indicated a satisfactory prediction efficiency of our model on patients’ OS (**Figure 3F**, 3-year AUC: 0.781, 5-year AUC: 0.780, 8-year AUC: 0.767). Moreover, the Kaplan–Meier survival curve showed that the patients in the high-risk group have a worse prognosis (**Figure 3G**). Meanwhile, time-dependent AUC showed that the risk score combined clinical features tend to have a better prediction efficiency than risk score (**Figure 3H**). The same trend and great prediction efficiency were also observed in the validation cohort (**Figures 3I-L**). Univariate and multivariate Cox analysis showed that the IRS is a risk factor independent of other clinical features (**Figure S4**).

### Clinical Correlation and Biological Role of the IRS Model

**Figure 4:**
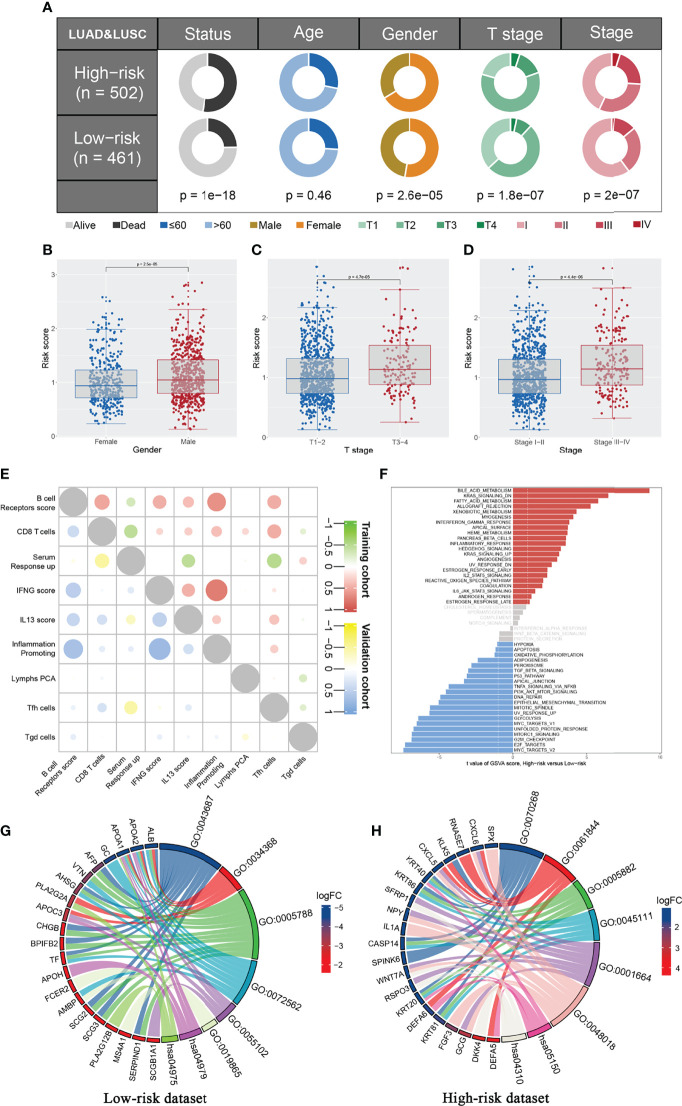
Clinical correlation and biological effect of IRS model. **(A)** The patients in high risk group have a higher percentage of more aggressive clinical features compared with the low risk patients, including T stage and clinical stage; **(B–D)** The IRS level between different group (Male vs. Female, T3-4 vs. T1-2, Stage III-IV vs. Stage I-II); **(E)** Co-expression immune terms of model immune terms; **(F)** GSEA analysis was performed to explore the biological pathway difference between high and low IRS group; **(G, H)** GO and KEGG pathway enrichment in low and high risk patients.

We further explored the clinical correlation and biological role of the IRS model to explain its prognosis effect. The result showed that patients in the high-risk group might have adverse clinical features, including T classification and clinical stage (**Figures 4A-D**). Interestingly, the male patients might have a higher IRS than the female patients, indicating their underlying immune microenvironment difference (**Figure 4B**). The co-expression relationship of nine model immune terms was visualized as a correlation coefficient heatmap to further explore their interactions (**Figure 4E**). GSEA demonstrated that in the high-risk group, the pathway of bile acid metabolism, KRAS signaling, fatty acid metabolism, xenobiotic metabolism, interferon-gamma response, inflammatory response, IL2-STAT5 signaling, and IL6-JAK-STAT3 signaling were activated, yet the hypoxia, P53 pathway, and DNA repair were downregulated (**Figure 4F** and **Figure S2**). GO and KEGG enrichment analysis showed that in the low-risk group, the terms post-translational protein modification (GO:0043687), protein–lipid complex remodeling (GO:0034368), endoplasmic reticulum lumen (GO:0005788), blood microparticle (GO:0072562), lipase inhibitor activity (GO:0055102), immunoglobulin binding (GO:0019865), cholesterol metabolism (hsa04979), and fat digestion and absorption (hsa04975) were enriched (**Figure 4G**). In the high-risk group, the terms cornification (GO:00702680), antimicrobial humoral immune response mediated by antimicrobial peptide (GO:0061844), intermediate filament (GO:0005882), intermediate filament cytoskeleton (GO:0045111), G protein-coupled receptor binding (GO:0001664), *Staphylococcus aureus* infection (hsa05150), and Wnt signaling pathway (has04310) were enriched (**Figure 4H**).

### The Correlation Between Genomic Features and IRS in NSCLC

**Figure 5:**
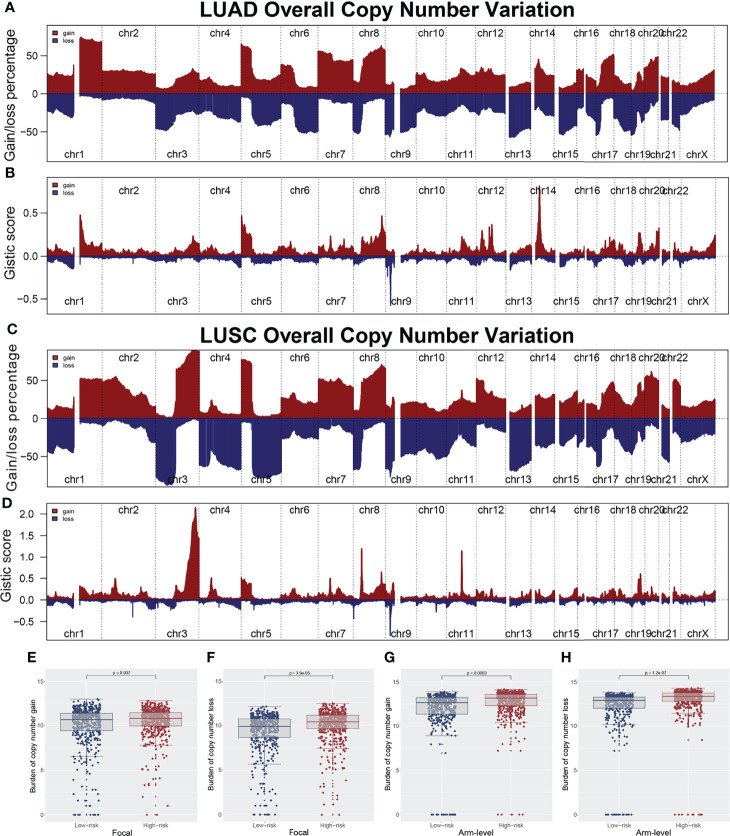
Copy number variation of TCGA-LUAD and TCGA-LUSC. **(A, B)** Copy number profiles (gain and loss) and gistic score of TCGA-LUAD; **(C, D)** Copy number profiles (gain and loss) and gistic score of TCGA-LUSC; **(E, F)** The copy number burden in focal level between low and high risk patients; **(G, H)** The copy number burden in arm level between low and high risk patients.

**Figure 6:**
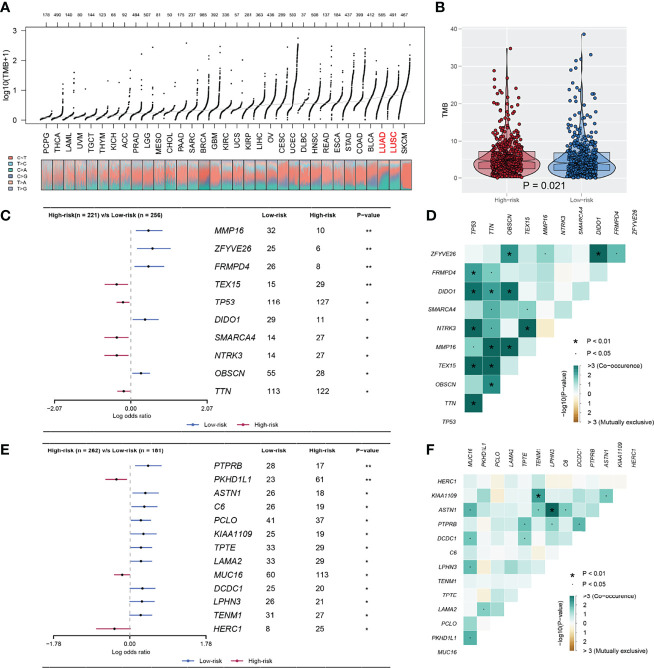
The TMB difference between high and low IRS group. **(A)** The overview of TMB in TCGA-pancancer; **(B)** A higher TMB level was observed in high risk patients compared with the low risk patients; **(C, D)** The mutated gene in TCGA-LUAD between high and low risk with P < 0.05; **(E, F)** The mutated gene in TCGA-LUSC between high and low risk with P < 0.05. *P < 0.05, **P < 0.01.

**Figure 7:**
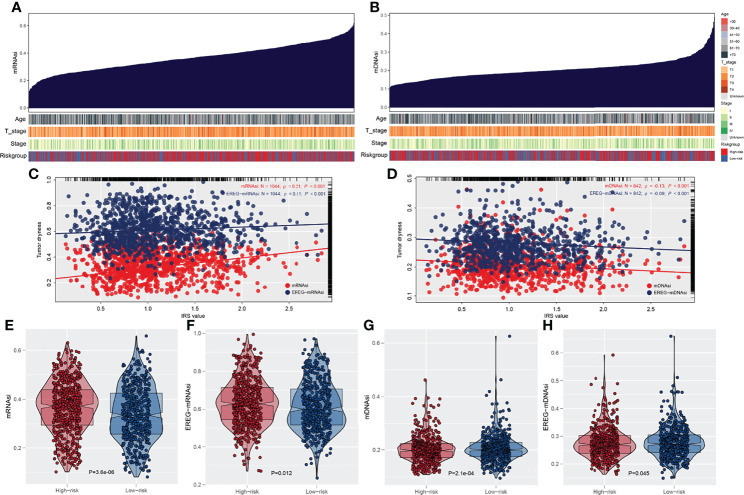
Association of tumor stemness with IRS. **(A)** Quantification of tumor stemness in RNA level; **(B)** Quantification of tumor stemness in DNA level; **(C, D)** The association between IRS and tumor stemness; **(E, F)** A higher degree tumor stemness in RNA level was observed in high risk patients; **(G, H)** A lower degree tumor stemness in RNA level was observed in high risk patients.

The overview of gene mutation of TCGA-NSCLC is shown in **Figure S3**. Then, the copy number profile in TCGA-LUAD and TCGA-LUSC patients, including gain/loss percentage and gistic score, was characterized (**Figures 5A–D**). The result showed that high-risk patients had a higher degree of genomic instability (**Figures 5E-H**). In detail, CNV burden analysis showed that high-risk patients had a higher level of focal and broad CNV burden in both gain and loss aspects (**Figures 5F-H**). The overview of the tumor mutation landscape of NSCLC is shown in **Figure 6A**. Moreover, higher TMB was also observed in the high-risk group (**Figure 6B**, *p* = 0.021). Furthermore, considering the intrinsic biological differences, we separately explored the mutant gene in TCGA-LUAD and TCGA-LUSC cohorts. The result showed that in the TCGA-LUAD cohort, TEX15, TP53, SMARCA4, NTRK3, and TTN were the most common mutated genes in the high-risk group, while the MMP16, ZFYVE26, FRMPD4, DIDO1, and OBSCN were the opposite (**Figure 6C**). In the TCGA-LUSC cohort, PKHD1L1, MUC16, and HERC1 were the most common mutated genes in the high-risk group, while the PTPRB, ASTN1, C6, PCLO, KIAA1109, TPTE, LAMA2, DCDC1, LPHN3, and TENM1 were the opposite (**Figure 6E**). The co-mutation relationship between these genes was then explored for the underlying interaction (**Figure 6D**, LUAD; **Figure 6F**, LUSC). Tumor stemness plays an important role in cancer progression. Therefore, we quantified the tumor stemness in the RNA and DNA level (**Figures 7A, B**). The result showed that risk score has a significantly positive correlation with mRNAsi and EREG-mRNAsi, but a negative correlation with mDNAsi and EREG-mDNAsi (**Figure 7C**, mRNAsi, *r* = 0.21, *p* < 0.001, EREG-mRNAsi, *r* = 0.11, *p* < 0.001; **Figure 7D**, mDNAsi, *r* = −0.13, *p* < 0.001, EREG-mDNAsi, *r* = −0.09, *p* < 0.001). Meanwhile, we found a higher mRNAsi and EREG-mRNAsi level in the high-risk group (**Figures 7E, F**). In addition, a lower mDNAsi and EREG-mDNAsi level was also observed in high-risk patients (**Figures 7G, H**).

### The IRS Model Is Closely Associated With Immunotherapy Response

**Figure 8:**
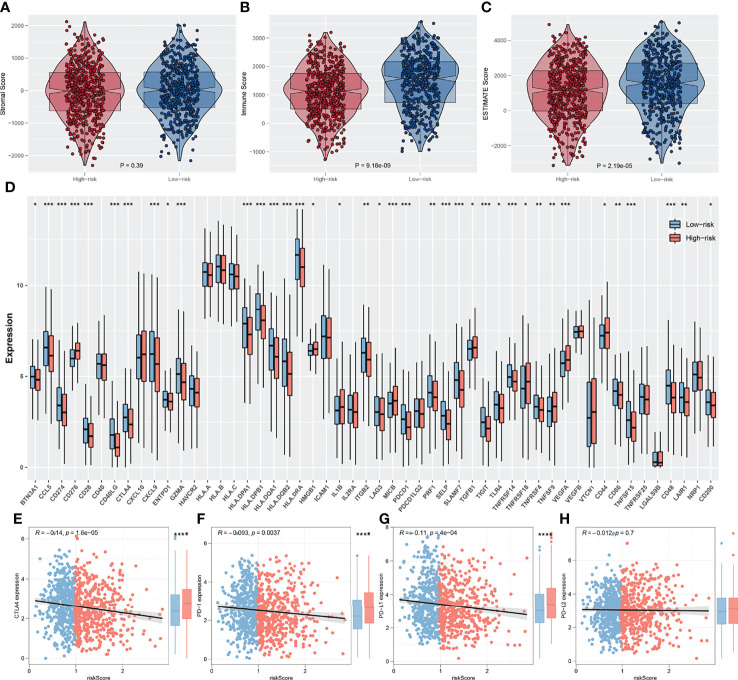
Exploration of the association of immune checkpoint genes with IRS. **(A–C)** The stromalscore, immunescore and estimatescore difference between high and low IRS group; **(D)** Significant differences were observed in multiple immune checkpoint genes between high and low IRS groups; **(E–H)** The difference of serval important immune checkpoint in high and low IRS patients. *P < 0.05, **P < 0.01, ***P < 0.001.

**Figure 9:**
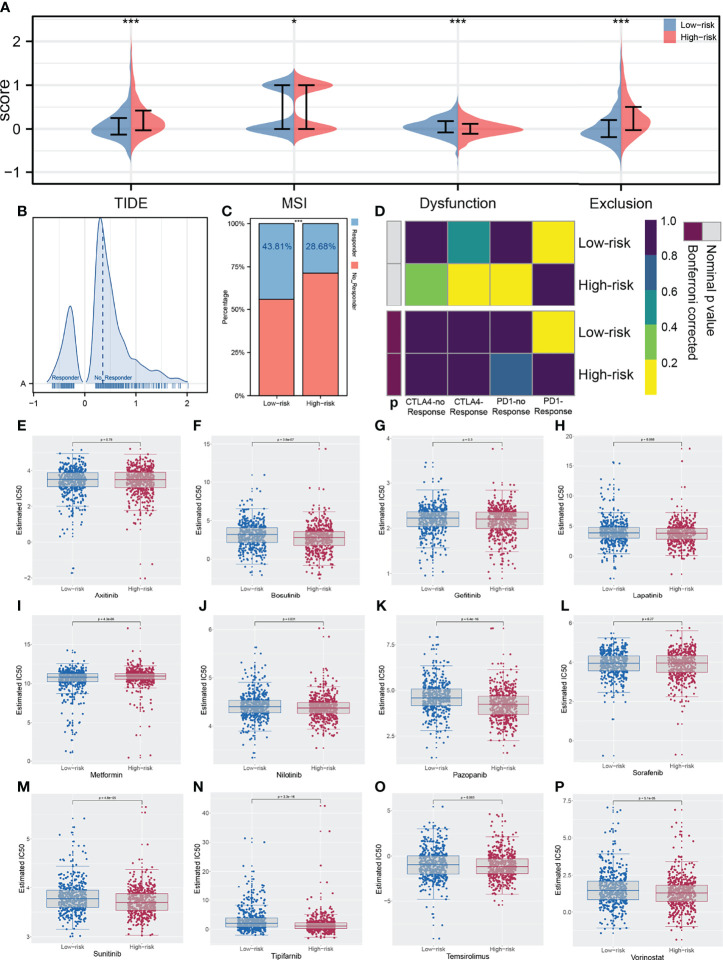
Correlation of IRS immunotherapy response rate and drug sensitivity. **(A)** TIDE analysis was performed in enrolled patients; **(B)** The patients with TIDE score < 0 were defined as immunotherapy responders, while > 0 were defined as immunotherapy non-responders; **(C)** The high risk group had a higher percentage of immunotherapy non-responders; **(D)** The low risk group might more sensitive to PD-1 immunotherapy; **(E–P)** Drug sensitivity analysis was performed to explore the underlying influence of IRS on common chemotherapy drugs. *P < 0.05, ***P < 0.001.

The ESTIMATE algorithm was used to quantify the tumor microenvironment score, including ESTIMATEScore, StromalScore, and ImmuneScore. No significant difference in StromalScore was found in high- and low-risk patients (**Figure 8A**). However, high-risk patients tend to have a lower ImmuneScore and ESTIMATEScore compared with low-risk patients (**Figures 8B, C**). Recently, immune checkpoints have gained a lot of interest among the scientific community. Therefore, we performed a correlation analysis between our IRS model and multiple checkpoint modules. The result showed a significant difference in several immune checkpoint modules between high- and low-IRS patients (**Figure 8D**). CTLA-4, PD-1, PD-L1, and PD-L2 were the immune checkpoint modules with great attention. We found that high-risk patients might have a higher CTLA-4, PD-1, and PD-L1 level, indicating the underlying immunotherapy response difference between low- and high-risk patients (**Figures 8E-H**). TIDE analysis was performed to explore the effect of IRS on immunotherapy response. The result showed that the patients in the high-risk group might have a higher TIDE score (**Figure 9A**). The patients with TIDE score < 0 were defined as immunotherapy responders, while patients with TIDE score > 0 were defined as immunotherapy non-responders (**Figure 9B**). We found that the high-risk group had a higher percentage of immunotherapy non-responders (**Figure 9C**). Based on the submap algorithm obtained from GenePattern, we found that patients in the low-risk group might be more sensitive to PD-1 immunotherapy (**Figure 9D**). Next, drug sensitivity analysis was performed to explore the underlying influence of IRS on common chemotherapy drugs (**Figures 9E–P**). The result showed that high-risk patients might be more sensitive to bosutinib, lapatinib, nilotinib, pazopanib, sunitinib, tipifarnib, temsirolimus, and vorinostat, yet resistant to metformin.

## Discussion

In recent years, PD-1/L1 inhibitors have changed the treatment landscape of advanced lung cancer patients. However, the relatively lower response rate and higher immune-related adverse occurrence rate are hardly satisfactory (16). Thus, it is necessary to explore another precise predictive biomarker for immunotherapy treatment. With the development of bioinformatics analysis, it is feasible for researchers to find another reliable biomarker in large populations.

In our study, we first enrolled 1,997 samples from six independent lung cancer cohorts that contained RNA-seq and clinical data. Then, the ssGSEA algorithm was performed to probe 54 immune terms in a combined expression profile. Furthermore, TCGA-LUAD combined with TCGA-LUSC samples were involved as a training group and other cohorts were classified as a validation group. Subsequently, nine prognostic-related immune terms were explored through univariate Cox regression analysis, LASSO analysis, and multivariate Cox regression analysis. We calculated a prognosis-related immune term signature called IRS based on these nine immune terms. According to the cutoff point, all patients were classified as high-risk and low-risk groups. Our study showed that these immune terms were significantly or marginally significantly associated with the prognosis in NSCLC patients. In addition, high IRS demonstrated aggressive clinical characteristics, worse prognosis, higher level of CNV burden, and higher tumor burden mutation and tumor stemness indices. In addition, the IRS model might effectively predict the response to immunotherapy and targeted therapy in NSCLC patients.

In our study, to enhance trustworthiness and credibility, we collected a total of 1,997 samples from six large lung cancer cohorts. Nine prognosis-associated immune terms were extracted after LASSO and Cox regression analysis. Meanwhile, these nine immune terms that included five protective factors and four risk factors were significantly or marginally significantly associated with the prognosis of lung cancer patients, implying a role in lung cancer progression. For example, Kalli et al. and Papageorgis et al. both indicated that IL13 could promote metastasis of breast cancer cells to lung tissue (17, 18). Moreover, IFN-γ and CD8+T cells serving as risk factors to prognosis could have surprised us because CD8+T cells and IFN-γ secretion were always thought as anti-tumor immune surveillance. We postulated that effector phenotype and exhausted phenotype CD8+T cells both counted through ssGSEA may be the major causes. Exhausted phenotype CD8+T cells represented a dysfunctional state, which negatively regulated the function of tumor cell killing (19). In parallel, IFN-γ might enhance endogenous PD-L1 expression and boost tumor metastasis in the tumor immune microenvironment (20). Therefore, it is critically important to facilitate the transformation towards anti-tumor phenotype (21). Recent studies also showed that intratumor-infiltrating B cells inhibited the early-stage lung cancer progression and predicted better immunotherapy response (22, 23). Moreover, the concordance index indicated that the combination of tumor stage and IRS signature could be a strong predictor of overall survival. These nine immune terms provided essential foundation to further explore the underlying tumor microenvironment and genomics information in NSCLC samples.

Our study showed that this novel model had good prognostic value in both training and validation groups. Then, we found that high IRS was correlated with advanced TNM stage, suggesting possible immune suppression. Pathway enrichment analysis displayed significant upregulation of bile acid metabolism, K-RAS signal, and fatty acid metabolism in high-IRS groups. Previous studies already showed that bile acid metabolism and fatty acid metabolism contributed to the tumor invasion (24, 25). Oncogenic K-RAS signaling that has been studied extensively promotes tumor progression in several cancers. Reck et al. indicated that K-RAS gene mutation was correlated with an immunosuppressive landscape through recruiting myeloid-derived suppressor cells (26). This could explain the different immunotherapy response between high- and low-IRS groups. P53 is a classical tumor-suppressive gene and downregulation of P53 was already verified to facilitate cancer progression in various cancers (27). GO and KEGG analysis demonstrated that programmed cell death signaling, G protein-coupled receptor signaling, humoral immune response, and WNT signaling pathway were mostly enriched pathways in high-IRS groups. Shen et al. reported that G protein-coupled estrogen receptor facilitated lung cancer cell metastasis through the NOTCH pathway (28). Our study indicated that high-IRS groups had worse clinical outcomes in combination with the abnormal activation of the mentioned pathways.

In addition, the high-IRS group demonstrated higher copy number alteration burden in the focal and arm level compared with the low-IRS group. This indicated that high-risk patients had a higher degree of genomic instability. It is generally accepted that genetic phenotypic differences caused by copy number alteration can facilitate tumor initiation and progression (29). Patients with a high level of CNV tend to be associated with decreased levels of immune cell infiltration landscape and elevated tumor proliferation ability (30). Meanwhile, CNVs were verified to be correlated with the prognosis of multiple cancers and the outcome of immunotherapy (31–33). A recent study showed that copy number alteration that can lead to genomic instability was indispensable in the transition from lung carcinoma *in situ* to invasive lung carcinoma (34). Gene mutation analysis revealed that patients with a high risk score possessed a higher TMB, indicating different potential response to immunotherapy. Our study was consistent with Liu et al., which showed that low TMB and low CNV were associated with better survival. In parallel, Liu et al. also revealed that those patients with high TMB and high CNV were relatively immunotherapy resistant (35). In addition, the probability of SMARCA4, NTRK3, and TEX15 mutation frequency was higher in high-risk LUAD groups. Concepcion et al. recently demonstrated that the SMARCA4 mutation type could decrease the chromatin accessibility and promote the progression of LUAD (36). NTRK was a rare genetic mutation in lung cancer and NTRK fusion inhibitor had already been used in clinical application (37). At the same time, there was a higher incidence of HERC1 and PKHD1L1 mutation in the LUSC group. The role of HERC1 and PKHD1L1 in LUSC was not reported and needed further validation. Furthermore, cancer stemness results showed that risk score has a positive correlation with mRNAsi and EREG-mRNAsi, but a weakly negative correlation with mDNAsi and EREG-mDNAsi (mRNAsi, *r* = 0.21, EREG-mRNAsi, *r* = 0.11; mDNAsi, *r* = −0.13, EREG-mDNAsi, *r* = −0.09). In the meantime, because mRNAsi was reflective of gene transcription expression, the high-IRS group exhibited higher tumor stemness index compared with the low-risk group (38). The study of Malta et al. showed that a higher tumor stemness index was highly correlated with tumor progression and lower PD-L1 expression level, which was shown in high-risk samples (39).

More importantly, high-IRS group patients had lower immune cell infiltration compared with the low-IRS group through the ESTIMATE algorithm (40). Our study also showed that the high-IRS group harbored a relative low PD-1, PD-L1, and CTLA-4 expression level, which usually means decreased immunotherapy efficacy. Then, results of TIDE and Subclass mapping algorithm showed that the patients with high IRS were not likely to benefit from immunotherapy. This conclusion was consistent with the previous finding. In addition, bosutinib, lapatinib, nilotinib, pazopanib, sunitinib, tipifarnib, temsirolimus, and vorinostat were all potential molecular targeted drugs for the treatment of high-IRS patients. Most of these drugs were already demonstrated to have an effect on lung cancer cells in recent years. Tan and his partners showed that bosutinib could repress the KRAS mutant lung cancer cells (41). Pazopanib blocked tumor growth and reduced the metastatic sites in lung cancer mouse models (42). Metformin acting as a traditional glucose-lowering agent could not inhibit the progression of lung cancer alone (43). Thus, further development of these agents is still warranted.

Nevertheless, some inevitable limitations should be noticed in our analysis. Firstly, this study was a retrospective analysis and simply using bioinformatics analysis were the largest weaknesses. Furthermore, a prospective sequencing data cohort is needed to support our IRS signature. Secondly, our study consisted only of a few Asian individuals available, which accounted for a small proportion of all cohorts. This situation might limit the clinical application of this IRS signature in China. Thirdly, the open-access data used for analysis were all at the mRNA level, and not at the protein level, which hardly reflects the real situation of lung cancer tissue.

## Conclusion

According to the prognosis-related immune terms quantified through ssGSEA, we established IRS signature to evaluate the outcome of NSCLC patients and respond to immunotherapy. Moreover, results indicated that high-IRS patients are inclined to bear increased genome instability, cancer CNV, and stemness indices, which mainly accounted for a dismal prognosis and a relatively low efficiency of immunotherapy. In addition, some targeted agents were identified including bosutinib, lapatinib, nilotinib, pazopanib, sunitinib, tipifarnib, temsirolimus, and vorinostat, which might reverse this adversity.

## Data Availability Statement

The datasets presented in this study can be found in online repositories. The names of the repository/repositories and accession number(s) can be found in the article/**Supplementary Material**.

## Author Contributions

Conception and design: YY. Administrative support: JW. Provision of study materials: FY. Data analysis and interpretation: WG. Manuscript writing: All authors. Final approval of manuscript: All authors.

## Funding

This study was supported by the Shanghai Key Laboratory of Clinical Geriatric Medicine, Shanghai Municipal Key Clinical Specialty (shslczdzk2801) and the Lung Cancer Diagnosis and Treatment Center (H1382).

## Conflict of Interest

The authors declare that the research was conducted in the absence of any commercial or financial relationships that could be construed as a potential conflict of interest.

## Publisher’s Note

All claims expressed in this article are solely those of the authors and do not necessarily represent those of their affiliated organizations, or those of the publisher, the editors and the reviewers. Any product that may be evaluated in this article, or claim that may be made by its manufacturer, is not guaranteed or endorsed by the publisher.

## Supplementary Material

Supplementary Figure 1 Kaplan-Meier survival curve of model immune terms, and the best cutoff of IRS in training and validation cohort. **(A)** Kaplan-Meier survival curve of model immune terms; **(B)** The best cutoff of IRS in training and validation cohort.

Supplementary Figure 2 GSEA enrichment analysis of IRS. **(A)** GSEA enrichment analysis of IRS model with all the GSEA gene set; **(B)** Representative pathway of each gene set.

Supplementary Figure 3 The overview of gene mutation of TCGA-NSCLC.

Supplementary Figure 4 Univariate and multivariate Cox regression analysis of IRS and clinical features. **(A)** Univariate Cox regression analysis of IRS and clinical features; **(B)** Multivariate Cox regression analysis of IRS and clinical features.
