# Supplementary figures and images for: Exploration of Novel Immunological Terms in Lung Cancer With Large Populations: Implications for Immunotherapy
Source: Front Immunol. 2022 Jun 30;13:924498. doi: 10.3389/fimmu.2022.924498 (PMC9280191; doi:10.3389/fimmu.2022.924498)

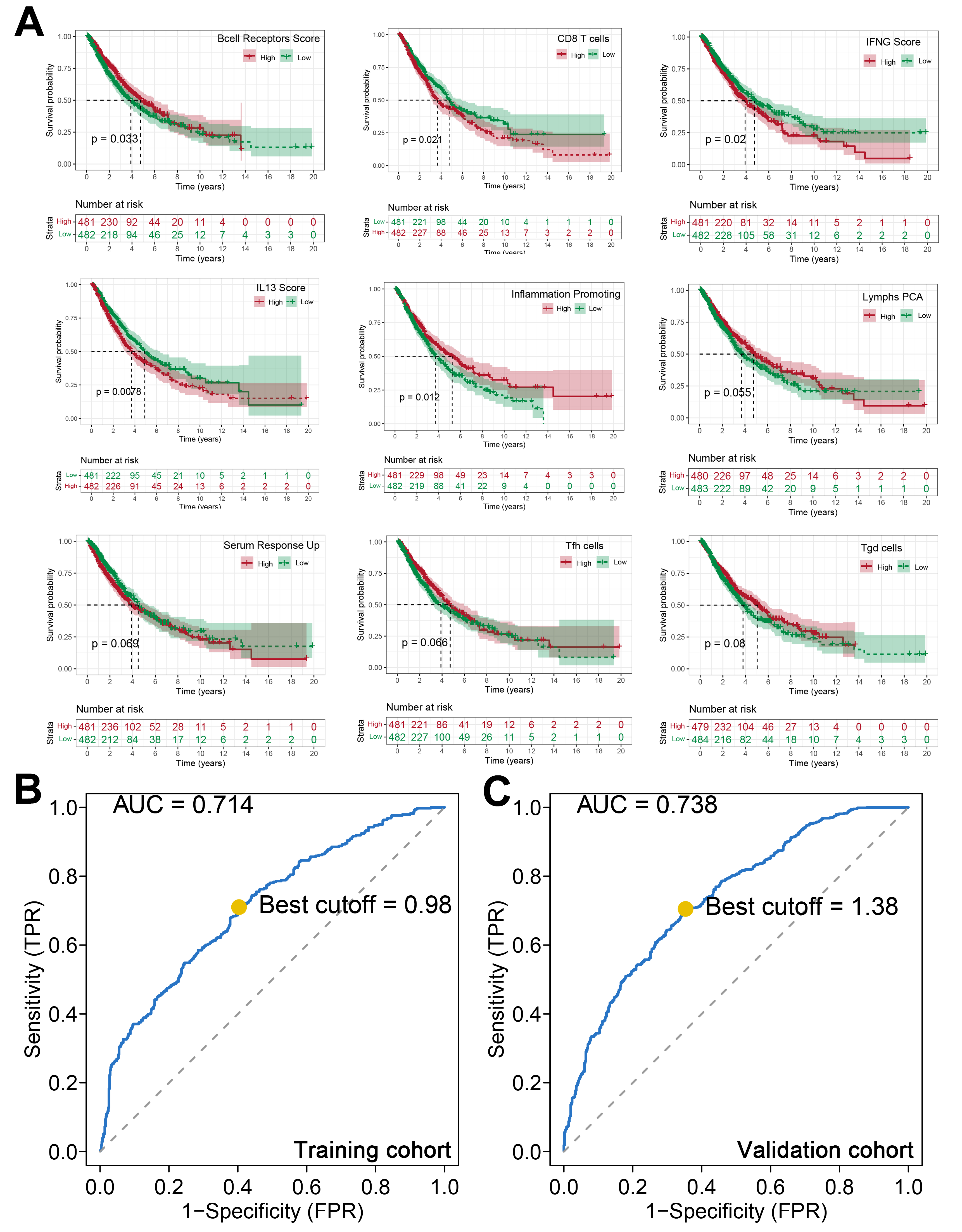

Supplement: Supplementary Figure 1 — Kaplan-Meier survival curve of model immune terms, and the best cutoff of IRS in training and validation cohort. (A) Kaplan-Meier survival curve of model immune terms; (B) The best cutoff of IRS in training and validation cohort. [file Image_1.tif]

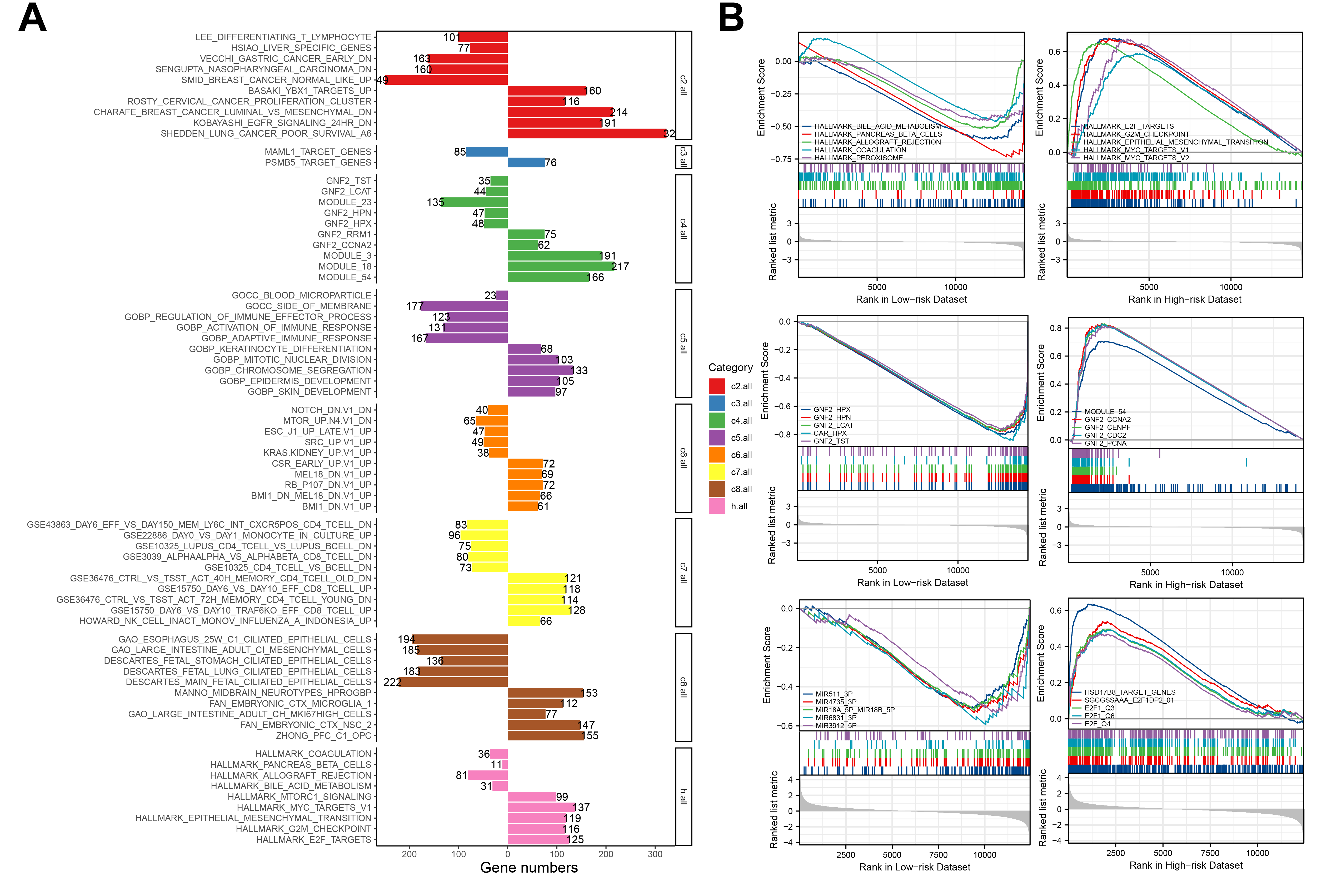

Supplement: Supplementary Figure 2 — GSEA enrichment analysis of IRS. (A) GSEA enrichment analysis of IRS model with all the GSEA gene set; (B) Representative pathway of each gene set. [file Image_2.tif]

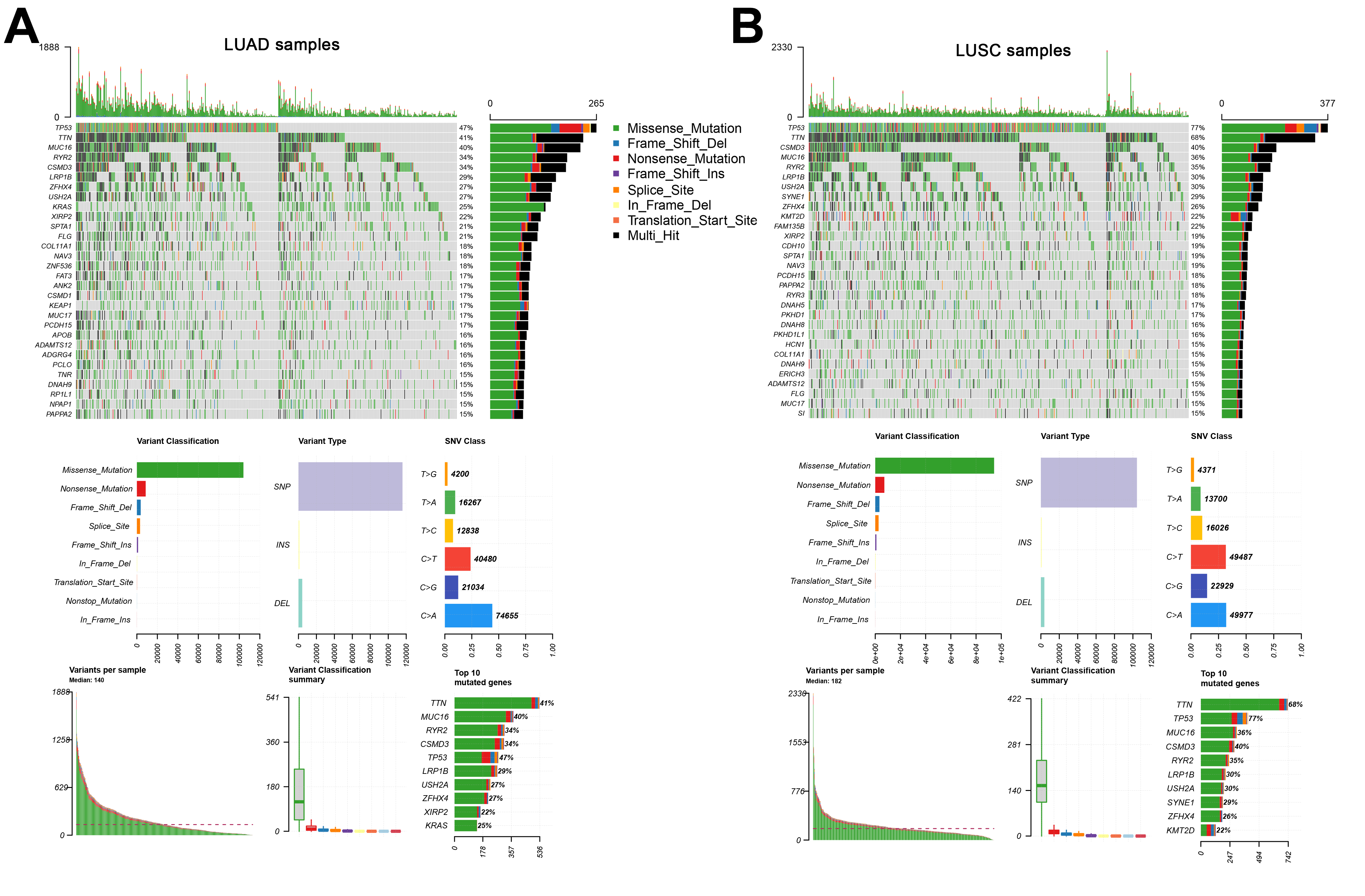

Supplement: Supplementary Figure 3 — The overview of gene mutation of TCGA-NSCLC. [file Image_3.tif]

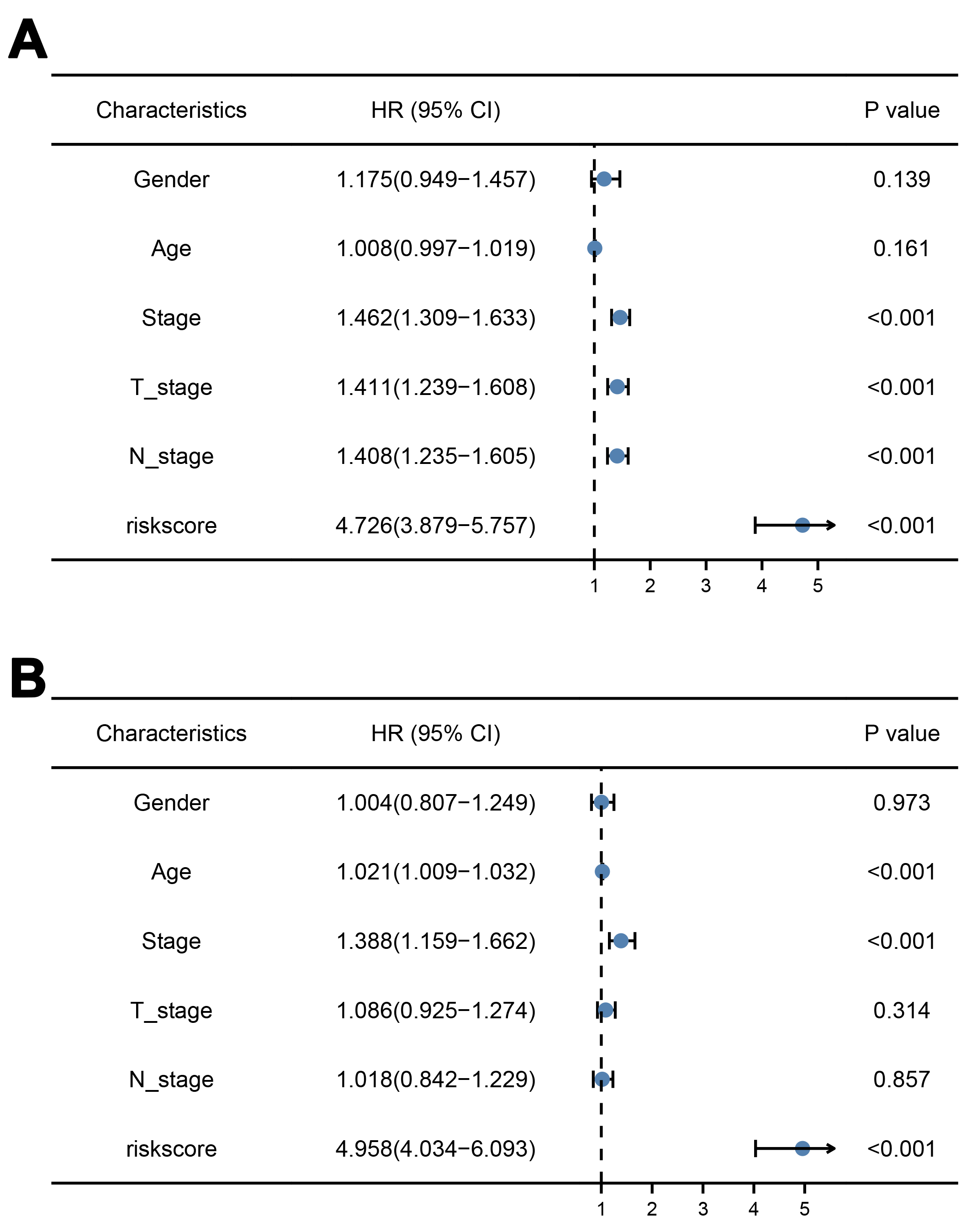

Supplement: Supplementary Figure 4 — Univariate and multivariate Cox regression analysis of IRS and clinical features. (A) Univariate Cox regression analysis of IRS and clinical features; (B) Multivariate Cox regression analysis of IRS and clinical features. [file Image_4.tif]
